# Adiponectin associates with markers of cartilage degradation in osteoarthritis and induces production of proinflammatory and catabolic factors through mitogen-activated protein kinase pathways

**DOI:** 10.1186/ar3512

**Published:** 2011-11-11

**Authors:** Anna Koskinen, Sami Juslin, Riina Nieminen, Teemu Moilanen, Katriina Vuolteenaho, Eeva Moilanen

**Affiliations:** 1 The Immunopharmacology Research Group, University of Tampere School of Medicine and Tampere University Hospital, Medisiinarinkatu 3, Tampere, FI-33014, Finland; 2 Coxa Hospital for Joint Replacement, Biokatu 6b, Tampere, FI-33520, Finland

## Abstract

**Introduction:**

Adiponectin is an adipokine that regulates energy metabolism and insulin sensitivity, but recent studies have pointed also to a role in inflammation and arthritis. The purpose of the present study was to investigate the association and effects of adiponectin on inflammation and cartilage destruction in osteoarthritis (OA).

**Methods:**

Cartilage and blood samples were collected from 35 male OA patients undergoing total knee replacement surgery. Preoperative radiographs were evaluated using Ahlbäck classification criteria for knee OA. Circulating concentrations of adiponectin and biomarkers of OA, that is, cartilage oligomeric matrix protein (COMP) and matrix metalloproteinase 3 (MMP-3), were measured. Cartilage samples obtained at the time of surgery were cultured *ex vivo*, and the levels of adiponectin, nitric oxide (NO), IL-6, MMP-1 and MMP-3 were determined in the culture media. In addition, the effects of adiponectin on the production of NO, IL-6, MMP-1 and MMP-3 were studied in cartilage and in primary chondrocyte cultures.

**Results:**

Plasma adiponectin levels and adiponectin released from OA cartilage were higher in patients with the radiologically most severe OA (Ahlbäck grades 4 and 5) than in patients with less severe disease (Ahlbäck grades 1 to 3). Plasma adiponectin concentrations correlated positively with biomarkers of OA, that is, COMP (*r *= 0.55, *P *= 0.001) and MMP-3 (*r *= 0.34, *P *= 0.046). Adiponectin was released by OA cartilage *ex vivo*, and it correlated positively with production of NO (*r *= 0.43, *P *= 0.012), IL-6 (*r *= 0.42, *P *= 0.018) and MMP-3 (*r *= 0.34, *P *= 0.051). Furthermore, adiponectin enhanced production of NO, IL-6, MMP-1 and MMP-3 in OA cartilage and in primary chondrocytes *in vitro *in a mitogen-activated protein kinase (MAPK)-dependent manner.

**Conclusions:**

The findings of this study show that adiponectin is associated with, and possibly mediates, cartilage destruction in OA.

## Introduction

Adiponectin belongs to the adipokine hormones, which were initially found to be synthesized by white adipose tissue and to control appetite and metabolism. Adiponectin was discovered in 1995 by Scherer *et al*. [1], and it was first named Acrp30 (adipocyte complement-related protein of 30 kDa). Adiponectin has been found to improve insulin sensitivity [2,3] and to have antiarthrogenic properties [4]. Interestingly, adiponectin has also been identified as a regulatory factor in inflammation and arthritis [5-8].

Adiponectin can be found in synovial fluid from osteoarthritis (OA) patients [9,10]. Tissues in the joint, including synovium, meniscus, osteophytes, cartilage, bone and fat, have been reported to produce adiponectin [10-12]. The biological effects of adiponectin are mediated through two adiponectin receptor subtypes, adiponectin receptor type 1 (AdipoR1) and adiponectin receptor type 2 (AdipoR2), which have been shown to be expressed in articular cartilage, bone and synovial tissue [13,14].

In arthritis models and in joint tissues, adiponectin has been postulated to have both pro- and anti-inflammatory effects. Adiponectin has been reported to increase the production of cartilage-degrading matrix metalloproteinase (MMP) enzymes, cytokines and prostaglandin E_2 _in chondrocytes and in synovial fibroblasts [11,14-19]. By contrast, intraarticularly injected adiponectin has been reported to mitigate the severity of collagen-induced arthritis in the mouse and to decrease immunohistochemically detected expression of TNF, IL-1 and MMP-3 [20]. Recently, high circulating adiponectin was found to correlate with cartilage degradation in patients with rheumatoid arthritis (RA) [21-23], although partly contradictory results have also been published [24,25].

Adiponectin has emerged as a regulator of immune responses and inflammatory arthritis [5-7], but its role in OA and cartilage degradation is controversial and, in many aspects, poorly known. The purpose of the present study was to investigate whether adiponectin is associated with radiographic severity or biomarkers of OA or with inflammatory and/or destructive factors released by cartilage samples obtained from OA patients. Since mitogen-activated protein kinase (MAPK) pathways have been proposed as therapeutic targets in OA [26,27], we decided also to study the possible involvement of these pathways in adiponectin-induced responses in OA cartilage.

## Materials and methods

### Patients and clinical studies

The patients in this study fulfilled the American College of Rheumatology classification criteria for OA [28]. Preoperative radiographs, blood samples and cartilage tissue were collected from 35 male patients with OA (means ± SEM: age = 69.5 ± 1.6 years, body mass index (BMI) = 29.3 ± 0.8 kg/m^2^) undergoing total knee replacement surgery at Coxa Hospital for Joint Replacement, Tampere, Finland. Radiographs were evaluated according to the Ahlbäck criteria, grades I to V, with grade V representing the most severe findings [29]. Plasma and serum samples were stored at -80°C until analyzed for cartilage oligomeric matrix protein (COMP), MMP-3 and adiponectin. Cartilage samples were processed as described below, and the amounts of adiponectin, NO, IL-6, MMP-1 and MMP-3 released by the cartilage *ex vivo *during a 42-hour incubation were measured as described below. The study was approved by the Ethics Committee of Tampere University Hospital and carried out in accordance with the Declaration of Helsinki. Written informed consent was obtained from the patients.

### Cartilage cultures

Leftover pieces of OA cartilage from knee joint replacement surgery were used. Full-thickness pieces of articular cartilage from femoral condyles, tibial plateaus and patellar surfaces showing macroscopic features of early OA were removed aseptically from subchondral bone with a scalpel, cut into small pieces and cultured in DMEM with GIBCO GlutaMAX-I supplemented with penicillin (100 U/ml), streptomycin (100 μg/ml) and amphotericin B (250 ng/ml) (all from Invitrogen/Life Technologies, Carlsbad, CA, USA) at 37°C in a humidified 5% carbon dioxide atmosphere.

Cartilage samples were incubated for 42 hours with or without adiponectin (recombinant human adiponectin produced in HEK cells; BioVendor Research and Diagnostic Products, Modřice, Czech Republic) and the MAPK inhibitors PD98059 (Erk1/2 inhibitor, 10 μM; Promega, Madison, WI, USA), SB220025 (p38 inhibitor, 0.5 μM; Calbiochem/Merck KGaA, Darmstadt, Germany) and SP600125 (JNK inhibitor, 10 μM; Calbiochem/Merck KGaA). The concentrations of adiponectin and MAPK inhibitors used in the experiments were based on preliminary experiments and studies previously carried out in our laboratory [30-32]. After the experiments, the cartilage explants were weighed and the results were expressed per milligram of cartilage. The culture media were kept at -20°C until analyzed.

### Primary chondrocyte experiments

The leftover pieces of OA cartilage were processed the same way as cartilage for cartilage cultures (see above). Cartilage pieces were washed with PBS, and chondrocytes were isolated by enzymatic digestion for 16 hours at 37°C in a shaker by using a collagenase enzyme blend (1 mg/ml Liberase TM Research Grade medium; Roche, Mannheim, Germany). Isolated chondrocytes were washed and plated on 24-well plates (1.5 × 10^5 ^cells/ml) in culture medium (DMEM with supplements; see above) containing 10% fetal bovine serum. Cells were treated with increasing concentrations of adiponectin (0.1 to 3 μg/ml) for 24 hours. The culture media were kept at -20°C until analyzed. Concentrations of NO, IL-6, MMP-1 and MMP-3 were determined in culture media as described below. To investigate MAPK activation (phosphorylation), cells were treated with adiponectin for 30 or 60 minutes and processed for Western blot analysis.

### NO production

Concentrations of nitrite, a stable metabolite of NO in aqueous solutions, were measured by Griess reaction [33].

### Measurement of adiponectin, COMP, MMP-1, MMP-3 and IL-6

Concentrations of adiponectin, COMP, MMP-1, MMP-3 and IL-6 in plasma, serum and/or medium samples were determined by performing ELISA with commercial reagents (adiponectin, MMP-1 and MMP-3: R&D Systems Europe Ltd, Abindgon, UK; COMP: BioVendor; IL-6: Sanquin, Amsterdam, The Netherlands).

### Western blot analysis

Western blot analysis was performed as previously described [34] using the following antibodies: rabbit anti-human pAb inducible nitric oxide synthase (iNOS), actin and c-Jun N-terminal kinase (JNK) antibodies, and horseradish-conjugated goat anti-rabbit immunoglobulin G antibody from Santa Cruz Biotechnology (Santa Cruz, CA, USA) and rabbit anti-human pAb p38, phospho-p38, phospho-JNK, extracellular signal-regulated kinase 1/2 (Erk1/2) and phospho-Erk1/2 antibodies from Cell Signaling Technology, Inc (Beverly, MA, USA).

### Statistical analysis

Data were analyzed using SPSS version 17.0 for Windows software (SPSS Inc, Chicago, IL, USA) and GraphPad InStat version 3.00 software (GraphPad Software, San Diego, CA, USA). The results are presented as means ± SEM unless otherwise indicated. Pearson's correlation analysis was carried out, and *r *values over +0.3 and under -0.3 were considered to indicate a correlation. Differences between groups were tested by one-way analysis of variance (ANOVA) or repeated-measures ANOVA, followed by Fisher's least significant difference test or the Bonferroni correction for multiple comparisons when appropriate. *P*-values less than 0.05 were considered significant. Standard multiple regression analysis was used to predict circulating biomarker levels (COMP and MMP-3) when adiponectin, age and BMI were set as independent variables. Nonstandardized regression coefficients (β) and coefficients of determination squared (*R*^2^) with the related *P*-values were calculated. Binary logistic regression was used to compute (BMI or age-adjusted) ORs for plasma adiponectin and adiponectin released by cartilage to predict the most severe radiographic findings (Ahlbäck grade 4 or 5 vs grades 1 to 3). A statistician was consulted regarding the statistical analysis.

## Results

### Correlation between plasma adiponectin and biomarkers of osteoarthritis

**Figure 1:**
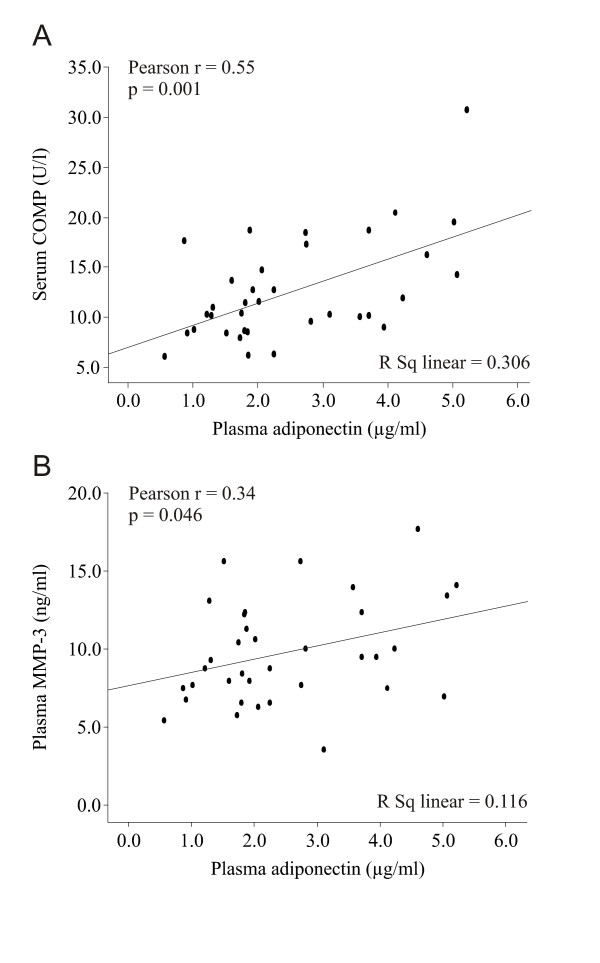
**Adiponectin correlates with biomarkers of OA**. Scatterplot showing positive correlations between plasma adiponectin and biomarkers of osteoarthritis (OA), serum cartilage oligomeric matrix protein (COMP) **(A) **and plasma matrix metalloproteinase 3 (MMP-3) **(B)**. *N *= 35 OA patients.

Thirty-five male OA patients were included in the study. Mean adiponectin concentration in plasma was 2.5 ± 0.2 μg/ml, and no correlation between plasma adiponectin and BMI was found (*r *= -0.15, *P *= 0.379). Interestingly, adiponectin correlated positively with the biomarkers of OA, that is, COMP (*r *= 0.55, *P *= 0.001) (Figure 1A) and MMP-3 (*r *= 0.34, *P *= 0.046) (Figure 1B), pointing to a possible connection between adiponectin and cartilage matrix degradation.

In multiple regression analysis, where serum COMP was set as a dependent variable and plasma adiponectin, age and BMI were set as predictive variables, adiponectin (β (that is, expected change in COMP with 1-U change in adiponectin) = 1.7; *P *= 0.010), but not BMI (β = -0.10, *P *= 0.566) or age (β = 0.14, *P *= 0.160), was a significant determinant of COMP (*R*^2 ^= 0.39 and *P *= 0.001 for model). Also, adiponectin was a significant determinant of MMP-3 when it was set alone as an independent variable (β = 0.85, *P *= 0.046; *R*^2 ^= 0.12 and *P *= 0.046 for model). Addition of BMI or age as independent variables did not improve the model (*R*^2 ^= 0.15, *P *= 0.070 or *R*^2 ^= 0.14, *P *= 0.089, respectively).

### Plasma adiponectin levels and radiographic severity of osteoarthritis

**Figure 2:**
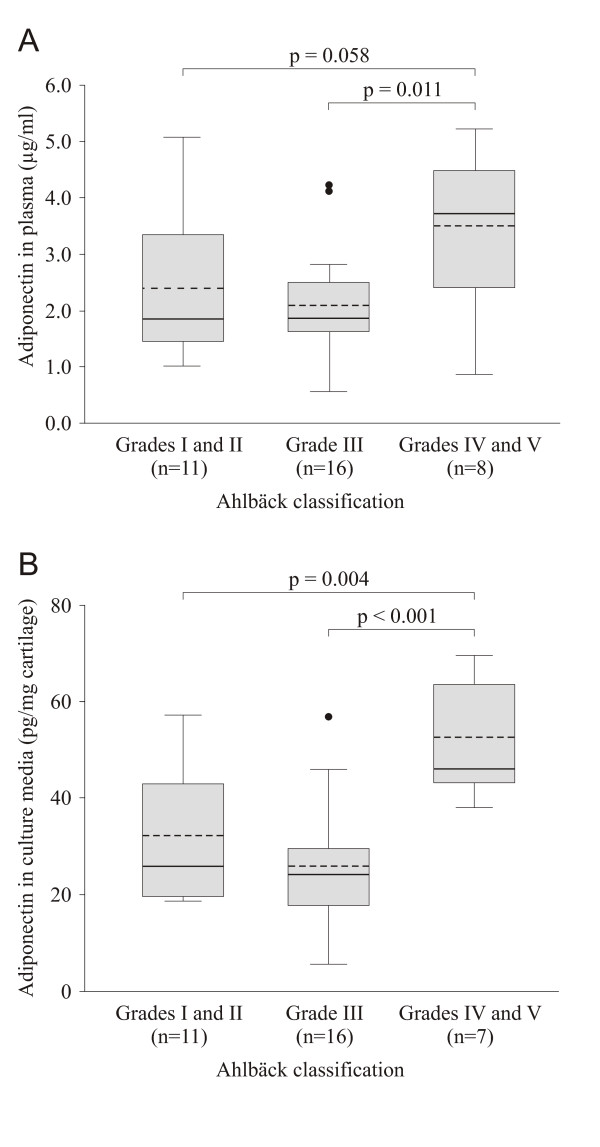
**Adiponectin levels in plasma and those released by cultured cartilage were higher in patients with radiologically more severe osteoarthritis**. Adiponectin levels in plasma **(A) **and the amount of adiponectin released by osteoarthritis (OA) cartilage samples into culture media **(B) **in OA patients classified according to the severity of knee OA evaluated using the Ahlbäck grading scale. Horizontal solid and dashed bars within the boxes represent the median and mean, respectively. Boxes represent the interquartile range. Lines outside boxes represent minimums and maximums. Outliers are indicated.

Preoperative radiographs of the knees were evaluated by Ahlbäck classification from grades 1 to 5, with grade 5 representing the most severe findings [29]. Grades 1 and 2, and 4 and 5 were combined to create more equally distributed subgroups. Mean plasma adiponectin concentrations were higher in the grades 4 and 5 group than in the grade 3 and grades 1 and 2 groups (Figure 2A), but there was no difference between the grades 1 and 2 group and the grade 3 group. There were no significant differences in age or BMI between the radiographic subgroups, but serum COMP was higher in the grades 4 and 5 group than in the grade 3 group and the grades 1 and 2 group (*P *= 0.012 and *P *= 0.006, respectively) (Table 1). Binary logistic regression analysis was used to further evaluate whether adiponectin is associated with the radiographic severity of OA (Ahlbäck grades 4 and 5 group vs Ahlbäck grades 1 to 3 group). When set alone in the model, plasma adiponectin and cartilage culture medium adiponectin, but not BMI or age, were significant explanatory factors of radiographic severity (Table 2). After adjusting for BMI, plasma adiponectin was a significant predictor of disease severity and almost statistically significant after adjusting for age (Table 2). Adiponectin measured in the cartilage culture media was a significant predictor of OA severity after controlling for age and BMI (Table 2).

**Table 1:** Plasma adiponectin levels and clinical characteristics of patients in radiographic subgroups

	Radiographic severity of osteoarthritis by Ahlbäck classification				
			
Patient characteristics	Grades 1 and 2 (*n *= 11)	Grade 3 (*n *= 16)	Grades 4 and 5 (*n *= 8)	Total (*N *= 35)	*P*-values
Adiponectin (μg/ml)	2.4 (0.4)	2.1 (0.2)	3.5 (0.5)	2.5 (0.2)	0.03
Age (years)	68.7 (3.0)	67.3 (2.6)	75.0 (2.0)	69.5 (1.6)	0.17
BMI (kg/m^2^)	28.7 (1.1)	29.8 (1.4)	29.0 (1.6)	29.3 (0.8)	0.84
COMP (U/L)	10.6 (1.1)	11.7 (1.0)	17.0 (2.4)	12.6 (0.9)	0.01
MMP-3 (ng/ml)	9.6 (1.1)	9.2 (0.6)	10.9 (1.5)	9.7 (0.6)	0.52

BMI = body mass index; COMP = cartilage oligomeric matrix protein; MMP-3 = matrix metalloproteinase 3. Values are means (SEM). *P*-values refer to the significance of the comparison of the three groups calculated by analysis of variance.

**Table 2:** Association of adiponectin and radiographic severity of osteoarthritis

	Ahlbäck grades 4 and 5			Ahlbäck grades 1 to 3		
	
Patient characteristics	Crude OR	95% CI	*P*-values	Adjusted OR	95% CI	*P*-values
Plasma adiponectin (μg/ml)	2.2	1.1 to 4.3	0.022	2.2^a^	1.1 to 4.4	0.022
				1.9^b^	0.9 to 3.8	0.090
Culture media adiponectin (pg/mg cartilage)	1.1	1.0 to 1.2	0.007	1.1^a^	1.0 to 1.2	0.007
				1.1^b^	1.0 to 1.2	0.016
BMI (kg/m^2^)	1.0	0.8 to 1.2	0.852			
Age (years)	1.1	1.0 to 1.3	0.078			

^a^Adjusted for BMI. ^b^Adjusted for age. Odds ratios are given for Ahlbäck grades 4 and 5 group vs grades 1 to 3 group.

### Production of adiponectin and inflammatory and/or degrading factors by osteoarthritis cartilage *ex vivo*

**Figure 3:**
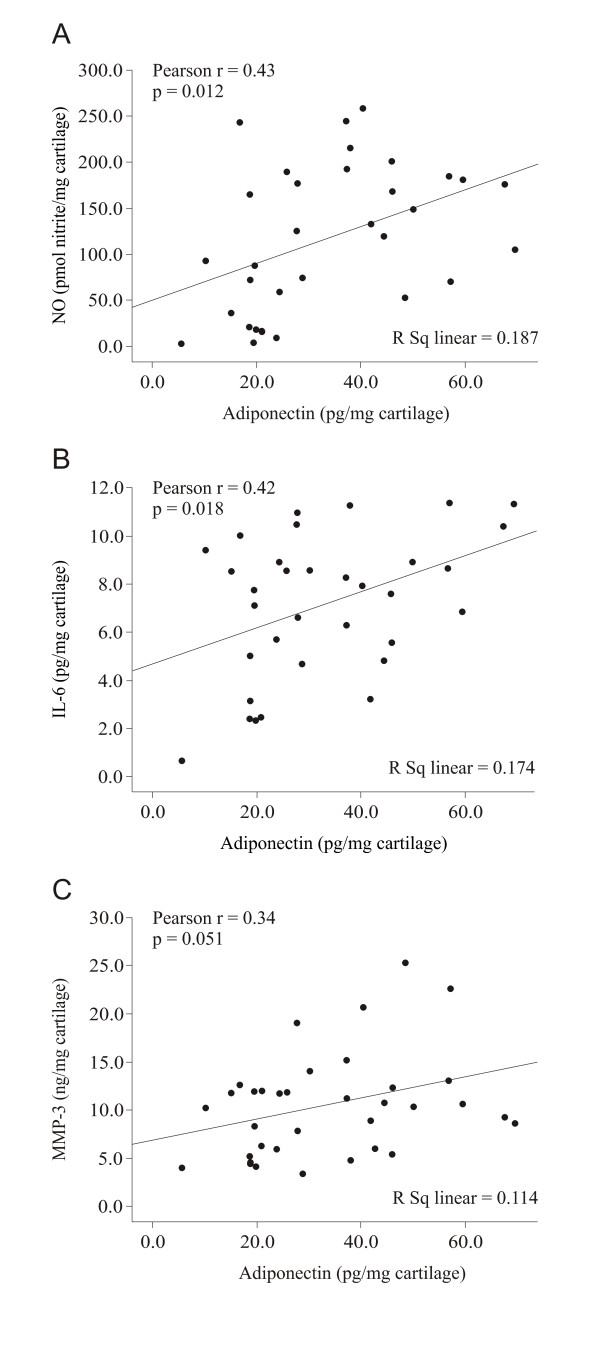
***Ex vivo *cartilage releases adiponectin and its concentration correlates with NO, IL-6 and MMP-3 production**. Scatterplots show positive correlations between adiponectin and NO **(A)**, IL-6 **(B) **and matrix metalloproteinase 3 (MMP-3) **(C) **released by osteoarthritis (OA) cartilage into the culture medium. Cartilage samples were collected from 35 OA patients undergoing knee replacement surgery.

Cartilage samples were obtained during joint replacement surgery from the same patients from whom the preoperative radiographs and the blood samples had been collected (see above), and tissue culture experiments were carried out. The amounts of adiponectin, NO, IL-6, MMP-1 and MMP-3 released from the cartilage into the culture medium during 42-hour incubation were measured. Adiponectin release was increased in patients with the radiographically most severe OA (Ahlbäck grades 4 and 5) as compared to patients in grades 1 and 2 and those in grade 3 (*P *= 0.004 and *P *< 0.001, respectively) (Figure 2B). Interestingly, adiponectin levels in the cartilage culture media correlated positively with those of NO (*r *= 0.43, *P *= 0.012) (Figure 3A), IL-6 (*r *= 0.42, *P *= 0.018) (Figure 3B) and MMP-3 (*r *= 0.34, *P *= 0.051) (Figure 3C), whereas no correlation between adiponectin and MMP-1 production was found (*r *= 0.17, *P *= 0.31).

### Effect of adiponectin on osteoarthritis cartilage and primary chondrocytes *in vitro*

**Figure 4:**
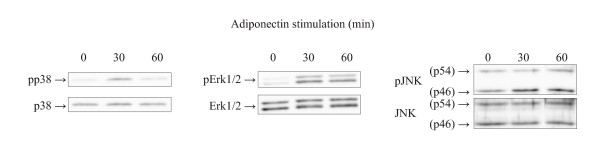
**Adiponectin induces activation of mitogen-activated protein kinases in human primary chondrocytes**. The effect of adiponectin (3 μg/ml) on mitogen-activated protein kinase (MAPK) phosphorylation in human primary chondrocytes obtained from patients with OA. The figure shows the results of a representative experiment which was repeated three times (that is, with cells from three donors) with similar results. MAPKs were determined by Western blot analysis at baseline and at 30 and 60 minutes after addition of adiponectin. Erk1/2 = extracellular signal-regulated kinase 1/2; JNK: c-Jun N-terminal kinase.

**Figure 5:**
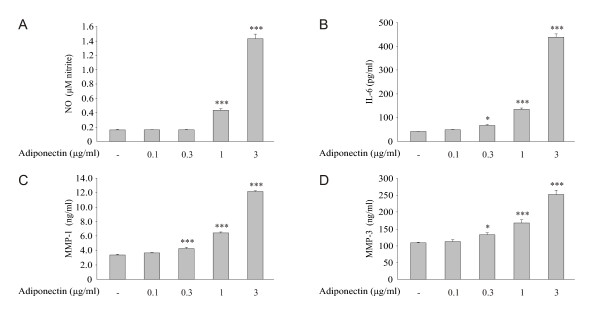
**Adiponectin enhances NO, IL-6, MMP-1 and MMP-3 production in human primary chondrocytes in a dose-dependent manner**. Chondrocytes obtained from patients with osteoarthritis (OA) were treated with increasing concentrations of adiponectin (0.1 to 3 μg/ml). Concentrations of NO **(A)**, IL-6 **(B)**, matrix metalloproteinase 1 (MMP-1) **(C) **and MMP-3 **(D) **were measured by the Griess reaction and ELISA in the culture medium after 24-hour incubation. The figure shows the results of a representative experiment which was repeated three times (that is, with cells from three donors) with similar results. Results are expressed as means ± SEM (*n *= 4). *P < 0.05 and ***P < 0.001 compared to the control sample.

**Figure 6:**
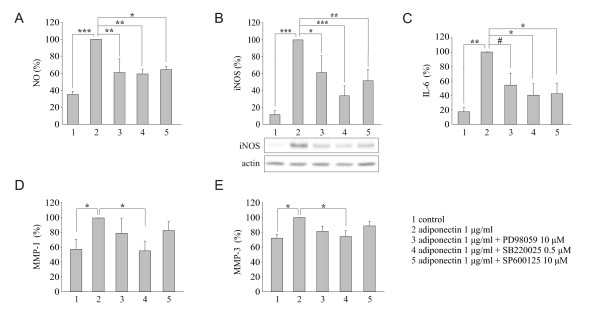
**Mitogen-activated protein kinase pathways are involved in adiponectin induced NO, IL-6, MMP-1 and MMP-3 production in osteoarthritis cartilage**. The effects of mitogen-activated protein kinase inhibitors on adiponectin-induced NO production **(A)**, inducible nitric oxide synthase (iNOS) expression **(B)**, IL-6 production **(C)**, matrix metalloproteinase 1 (MMP-1) production **(D) **and MMP-3 production **(E) **in human osteoarthritis cartilage. Cartilage explants were incubated for 42 hours with adiponectin (1 μg/ml) and the inhibitor indicated. Samples were collected from six patients in **(A) **and **(B) **and from five patients in **(C) **through **(E)**. Results are expressed as means ± SEM. ^#^P < 0.1, *P < 0.05, **P < 0.01 and ***P < 0.001 compared to explants treated with adiponectin alone. PD98059 = extracellular signal-regulated kinase 1/2 (Erk1/2) inhibitor; SB220025 = p38 inhibitor; SP600125 = c-Jun N-terminal kinase (JNK) inhibitor. The inhibitor concentrations used were based on previous studies in our laboratory [30-32].

To further evaluate the role of adiponectin in OA, we studied the effect of this adipokine on MAPK phosphorylation (that is, activation) and on NO, IL-6, MMP-1 and MMP-3 production in primary chondrocytes from OA patients. Adiponectin treatment resulted in time-dependent phosphorylation of p38, Erk1/2 and JNK in primary OA chondrocytes that was obvious within 30 minutes and decreased toward baseline by 60 minutes (Figure 4). Adiponectin also enhanced NO, IL-6, MMP-1 and MMP-3 production in primary OA chondrocytes in a dose-dependent manner (Figure 5). Because cartilage matrix is an important regulator of chondrocyte metabolism, we wanted to investigate the effects of adiponectin on OA cartilage in tissue culture. Owing to the limited amount of tissue available for the experiments, one concentration of adiponectin (1 μg/ml) was selected based on the cell culture studies (Figure 5) and previously published data on adiponectin levels in OA synovial fluid [9,10,12,35]. Adiponectin enhanced NO, IL-6, MMP-1 and MMP-3 production and iNOS expression in OA cartilage culture, and their production was suppressed by the p38 MAPK inhibitor SB220025 (0.5 μM) (Figure 6). In addition, the Erk1/2 inhibitor PD98059 (10 μM) and the JNK inhibitor SP600125 (10 μM) inhibited adiponectin-induced production of IL-6 and NO, as well as expression of iNOS, in a statistically significant manner, whereas their effect on MMP-1 and MMP-3 was smaller and did not reach statistical significance (Figure 6).

## Discussion

Adiponectin is found in OA joints, and proinflammatory and catabolic effects have been reported [9,10,13-19]. On the basis of our findings of the present study, we show for the first time that the circulating adiponectin concentrations correlate positively with the levels of the widely used biomarkers of OA, that is, COMP and MMP-3, and that plasma adiponectin levels, as well as adiponectin levels released by cultured cartilage, are associated with the radiographic severity of OA. Interestingly, the amount of adiponectin released by OA cartilage *ex vivo *also correlated positively with the production of inflammatory mediators NO and IL-6 and with the matrix-degrading enzyme MMP-3. Furthermore, adiponectin, when added at physiological concentrations to cultures of intact human OA cartilage or primary OA chondrocytes, enhanced the production of inflammatory and/or destructive factors NO, IL-6, MMP-1 and MMP-3. These findings suggest that adiponectin is associated with cartilage matrix degradation and has a role in the pathogenesis or as a biomarker in OA.

In the present study, we measured circulating levels of COMP and MMP-3 to evaluate the degree of ongoing cartilage destruction in OA [36]. The level of serum COMP has been shown to correlate with the grade of OA assessed by the radiological score [37], which we also observed in this study. Also, the concentrations of MMP-3 have been reported to associate with joint space narrowing [38]. The present results demonstrate for the first time that plasma adiponectin levels correlate with COMP and MMP-3, suggesting an association between adiponectin and the degree of ongoing cartilage matrix degradation.

We also found that plasma adiponectin levels and adiponectin amounts released by cultured OA cartilage *ex vivo *were higher in patients with radiographically advanced OA (grades 4 and 5 according to the Ahlbäck classification system) than in patients with less severe disease (Ahlbäck grades 1 to 3). This suggests that adiponectin is associated with cartilage degradation in patients with OA. Our results are supported by the recent studies by Ebina *et al*. [21], Giles *et al*. [22] and Klein-Wieringa *et al*. [23], who showed that circulating adiponectin correlates with joint erosions in RA patients. Additional support for our findings is provided by the study of Laurberg *et al*. [39], who reported elevated plasma adiponectin concentrations in OA patients as compared to healthy controls. In our study, adiponectin released by cultured cartilage also correlated positively with NO, IL-6 and MMP-3 production in the cartilage.

Two recent studies have reported somewhat different findings on the association between plasma adiponectin levels and radiographic findings. In the study by Honsawek *et al*. [24], adiponectin concentrations in plasma and synovial fluid were lower in patients with more severe knee OA measured according to the Kellgren-Lawrence Grading Scale. After they adjusted for gender, age and BMI, their plasma findings became nonsignificant, but the differences between adiponectin levels in synovial fluid within the radiographic groups remained significant. Most of their patients were female, which may at least partly explain the differences between their results and ours. Also, the two different radiographic scaling systems emphasize different findings. We chose to use Ahlbäck grading, since it tends to divide the end-stage OA patients less roughly than the Kellgren-Lawrence Grading Scale, as reported by, for example, Petersson *et al*. [40]. Accordingly, most (80%) of our patients were scaled into the most severe Kellgren-Lawrence grade (grade 4). We included only male patients in the present study because gender is likely to be a confounding factor.

A study by Yusuf *et al*. [25] revealed that higher levels of plasma adiponectin decreased the risk for hand OA progression during a 6-year follow-up period as measured by radiographic changes. The findings of that study appear to be somewhat contradictory to our results and to those reported by the other research groups mentioned above. The differences may be explained by many factors, including different methodologies used to measure adiponectin, differences in patient characteristics and study protocols, gender differences (most of the patients in the study by Yusuf *et al*. were women) and possibly even by differences in the pathophysiologies of hand and knee OA. It is also possible that the significance of adiponectin varies according to the phase and severity of the OA process. It is noteworthy that all our patients had advanced OA and were undergoing joint replacement surgery. This made it possible to obtain simultaneous cartilage and blood samples. Lack of patients with less severe OA, however, limits our ability to generalize the results to milder cases.

An inverse relationship between adiponectin levels and BMI, especially visceral fat, has been reported in studies in which an endocrinological approach was used [41]. However, no correlation between adiponectin and BMI was found in several recent clinical studies in which patients with OA or RA were investigated [35,39,42]. This was also the case within our group of OA patients. This may be explained by the fact that circulating concentrations of adiponectin can be regulated by various hormonal, nutritional or pharmacological factors and that adiponectin is produced not only by white adipose tissue but by other tissues as well [41]. The question remains open whether there is such a systemic factor that affects the adiponectin levels in patients with arthritis or whether the joint disease itself rather than BMI might be a greater explanatory factor in defining the adiponectin levels in these patients.

As the clinical data suggest that the amount of adiponectin released by cartilage is related to the severity of cartilage degradation, we decided to study its possible mechanisms in OA cartilage. In agreement with recent findings [11,14-16,18], we found that adiponectin stimulated human OA cartilage and primary OA chondrocytes to produce NO, IL-6, MMP-1 and MMP-3, which are proinflammatory and catabolic mediators in OA [43-50]. In agreement with these findings, adiponectin was very recently reported to increase the production of chemokine IL-8 in human chondrocytes [19]. MAPK inhibitors are under development for treatment of OA [26,27], and MAPK pathways have been reported to be activated by adiponectin [51,52]. Therefore, we studied whether MAPK signaling pathways are also activated by adiponectin in articular chondrocytes and whether they might mediate adiponectin's effects on NO, IL-6, MMP-1 and MMP-3 production. Adiponectin was found to activate the kinases p38, JNK and Erk1/2 at physiologically relevant concentrations. The p38 inhibitor decreased the production of all factors studied in a statistically significant manner, whereas Erk1/2 was involved in adiponectin-induced iNOS expression and NO production and JNK was involved in NO, iNOS and IL-6 production. These results, together with recently published findings [14,18], show that MAPKs, especially p38, are significant pathways in adiponectin signaling in chondrocytes. Also, MAPK inhibitors are likely to attenuate adiponectin-induced gene expression in OA cartilage.

Adipokines, that is, hormones secreted by adipose tissue, have emerged as important modulating agents, not only in energy metabolism and appetite but also in the immune system and inflammation [53], and they are likely to have a role in mediating the connection between obesity and chronic inflammatory diseases. The actions of adiponectin, leptin, resistin and other, less studied adipokines in OA and other rheumatic diseases have recently been reviewed by Gómez *et al*. [7] and by Neumann *et al*. [8]. The most studied adipokine in the pathophysiology of arthritis is leptin, which has been proven to have proinflammatory and catabolic roles in OA [8,19,54-58]. Knowledge about adiponectin in joint diseases has accumulated only lately. The present results, together with those described in the other recent reports, strongly suggest a proinflammatory and catabolic role for adiponectin in OA and RA cartilage.

## Conclusions

We found that adiponectin was associated with markers and signs of cartilage degradation, that is, with circulating concentrations of COMP and MMP-3 and with radiographic severity of OA. Adiponectin was released by OA cartilage *ex vivo*, and it correlated with production of NO, IL-6 and MMP-3, which are important mediators in the pathogenesis of OA. Subsequent *in vitro *studies demonstrated that adiponectin, when added to the culture media, enhanced the production of NO, IL-6, MMP-1 and MMP-3 in OA cartilage and primary OA chondrocytes. Adiponectin also activated p38, Erk1/2 and JNK in chondrocytes, and the adiponectin-induced production of NO, IL-6, MMP-1 and MMP-3 were mediated by MAPKs, especially by p38. These findings strongly suggest that adiponectin is involved in the pathogenesis of joint inflammation and cartilage destruction in OA and may be a target for disease-modifying drug development.

## Abbreviations

BMI: body mass index; COMP: cartilage oligomeric matrix protein; DMEM: Dulbecco's modified Eagle's medium; ELISA: enzyme-linked immunosorbent assay; Erk1/2: extracellular signal-regulated kinase 1/2; HEK cells: human embryonic kidney cells; IL: interleukin; iNOS: inducible nitric oxide synthase; JNK: c-Jun N-terminal kinase; MAPK: mitogen-activated protein kinase; MMP: matrix metalloproteinase; NO: nitric oxide; OA: osteoarthritis; pAb: polyclonal antibody; PBS: phosphate-buffered saline; RA: rheumatoid arthritis; TNF: tumor necrosis factor.

## Competing interests

The authors declare that they have no competing interests.

## Authors' contributions

AK, SJ and KV were involved in the conception and design of the study, the laboratory analyses, calculation of the results and interpretation of the data, and they drafted the manuscript. RN was involved in the conception and design of the study, the laboratory analyses, the interpretation of the data and revising the manuscript. TM was involved in the conception and design of the study, selecting the patients, acquiring the patient samples, the interpretation of the data and revising the manuscript. EM was involved in the conception and design of the study, the interpretation of the data and writing the manuscript. All authors approved the final version of the manuscript.
